# Carbonyl Activation by Selenium‐ and Tellurium‐Based Chalcogen Bonding in a Michael Addition Reaction

**DOI:** 10.1002/chem.201905057

**Published:** 2020-01-21

**Authors:** Patrick Wonner, Tim Steinke, Lukas Vogel, Stefan M. Huber

**Affiliations:** ^1^ Fakultät für Chemie und Biochemie Ruhr-Universität Bochum Universitätsstraße 150 44801 Bochum Germany

**Keywords:** carbonyl activation, chalcogen bonding, chalcogens, Lewis acid catalysis, non-covalent organocatalysis

## Abstract

In the last years the use of chalcogen bonding—the noncovalent interaction involving electrophilic chalcogen centers—in noncovalent organocatalysis has received increased interest, particularly regarding the use of intermolecular Lewis acids. Herein, we present the first use of tellurium‐based catalysts for the activation of a carbonyl compound (and only the second such activation by chalcogen bonding in general). As benchmark reaction, the Michael‐type addition between *trans*‐crotonophenone and 1‐methylindole (and its derivatives) was investigated in the presence of various catalyst candidates. Whereas non‐chalcogen‐bonding reference compounds were inactive, strong rate accelerations of up to 1000 could be achieved by bidentate triazolium‐based chalcogen bond donors, with product yields of >90 % within 2 h of reaction time. Organotellurium derivatives were markedly more active than their selenium and sulphur analogues and non‐coordinating counterions like BAr^F^_4_ provide the strongest dicationic catalysts.

## Graphical abstract

**Make it active**! Tellurium‐based dicationic chalcogen bonding organocatalysts have been used to activate a carbonyl compound in the Michael addition reaction between indole derivatives and *trans*‐crotonophenone. Up to 1000‐fold rate accelerations were observed compared to the corresponding iodinated halogen bond donor. Several comparison experiments were performed to rule out other modes of activation than chalcogen bonding.

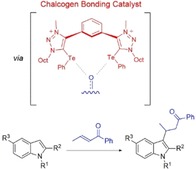

Chalcogen bonding^1^ denotes the attractive interaction between electrophilic chalcogen centers and Lewis bases. Its use in noncovalent organocatalysis—which is so far dominated by hydrogen bonding^2^—is an emerging topic.^3^ Compared to classical hydrogen‐based Lewis acids, chalcogen bonding compounds possess at least two features which make them valuable for such applications: a) an interaction angle of roughly 180° and b) the possibility to fine‐tune the activity of the catalyst by various means, including structural modifications of the backbone and the chalcogen atom.^4^ In addition, previous studies have shown that chalcogen‐bonding‐based catalysts can be superior in activity to ones based on hydrogen bonding or halogen bonding.^5^ So far, chalcogen bonding was mostly applied in solid state investigations and supramolecular chemistry.^6^ In organic synthesis, its intramolecular use to rigidify chiral selenenylation reagents had already been established in the mid‐1990s by Tomoda and Wirth.^7^ In contrast, intermolecular chalcogen bonding in solution has only been studied systematically in the last few years in the form of studies on anion binding and transport.^8^ Examples involving noncovalent organocatalysis still remain underrepresented.^9^

In 2017, first such cases employing neutral sulphur‐based or cationic selenium‐based catalysts were reported by Matile^10^ and our group,5a with the reactions involving the reduction of quinolines and an S_N_1‐based carbon–carbon bond formation, and similar ones were being investigated later on.5b, 11 Recently, our group could confirm the superior performance of cationic chalcogen bonding catalysts versus neutral ones in a direct comparison,^12^ and we also reported the first activation of a nitro group,5c using tellurium‐based dicationic catalysts. Also in 2019, Wang et al. described the use of bidentate selenophosphonium compounds as catalysts in a multicomponent reaction involving several carbonyl species, which likely constitutes the first activation of this functional group by chalcogen bonding.^13^ This, however, is still the only report on this issue, and there is no reported case on the activation of a carbonyl derivative in a “simple” two‐component transformation. Also, organotellurium compounds have not yet been employed as catalysts in this case, even though chalcogen bonding theory would predict them to be stronger in Lewis acidity.^14^

Herein, we present the first example, and as an appropriate test reaction we focused on the activation of an α,β‐unsaturated carbonyl compound in the Michael‐type addition reaction between 1‐methylindole (**1**) and *trans*‐crotonophenone (**2**) (Scheme 1). This reaction was chosen as a) it can be simply monitored by ^1^H NMR spectroscopy, b) there is virtually no background reactivity in absence of any activating agent at room temperature (Table 1, entry 1) and c) this type of reaction has already been activated by a related “unconventional” non‐covalent interaction, halogen bonding.^15^ In contrast to this earlier report, however, indole is here replaced by 1‐methylindole to avoid complications arising from the interference of the acidic N‐proton in the reaction mechanism.

**Scheme 1:**
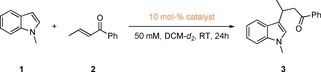
Benchmark reaction between 1 equivalent of 1‐methylindole (**1**) and 1 equivalent of *trans*‐crotonophenone (**2**) with various chalcogen bond donors and reference compounds as catalyst candidates.

**Table 1:** Performance of the reference compounds in the reaction between indole **1** and carbonyl compound **2**.

Entry	Catalyst	Loading [mol %]	Yield of **3** [%]^[b,c,d]^
1	–	–	<5
2	**4** ^I‐BArF4^	10	<5
3	**4** ^H‐BArF4^	10	<5
4	S	20^[a]^	<5
5	Se	20^[a]^	<5
6	Te	20^[a]^	<5
7	**5** ^S^	10	<5
8	**5** ^Se^	10	<5
9	**5** ^Te^	10	<5
10	**6** ^SPh^	20^a^	<5
11	**6** ^SePh^	20^a^	<5
12	**6** ^TePh^	20^a^	<5

[a] 20 mol % catalyst were used to ensure the same number of potential Lewis acidic centers as in bidentate catalysts. [b] ^1^H NMR yields of compound **3** after 24 h reaction time (with TES as internal standard). [c] Averaged values of at least two measurements. [d] No indication of catalyst decomposition was observed in any case.

As core structures for the catalyst candidates, we focused on bis(triazolium)benzene derivatives **4** (Figure 1), which had generated very active catalysts in our recent investigation on a nitro‐Michael reaction.5c Next to a variation of the chalcogen centers, the influence of the counter anion for the tellurium and selenium compounds (Figure 1, **4** ^Te‐Z^ and **4** ^Se‐Z^) was also of key interest, as studies on this issue are still quite rare.5c

**Figure 1:**
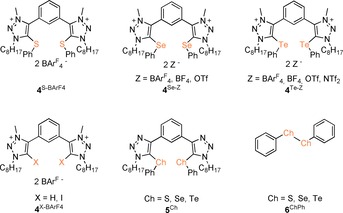
Overview of all tested chalcogen bond donors and reference compounds in the reaction between indole **1** and carbonyl compound **2**.

Prior to this, though, several reference compounds (**4** ^X‐BArF4^, **5** ^Ch^, **6** ^ChPh^; Figure 1) were tested to rule out any activation other than chalcogen bonding. First, the iodinated (**4** ^I‐BArF4^) and the non‐functionalized (**4** ^H‐BArF4^) analogues of chalcogen bond donors **4** ^Ch‐Z^ were employed with a catalyst loading of 10 mol %. Even though **4** ^I‐BArF4^ constitutes a relatively strong halogen bond donor, derivatives of which had been used successfully as Lewis acids before,^16^ both compounds showed only little activity with less than 5 % yield of compound **3** (Table 1, entries 2 and 3).

The same result was obtained for elemental sulphur, selenium and tellurium as potential catalysts even when 20 mol % were used (Table 1, entries 4–6).

Several non‐charged organochalcogen compounds were also inactive, namely the non‐alkylated precursors **5** ^Ch^ (which should be much weaker chalcogen bond donors; Figure 1) and the dichalcogenides **6** ^ChPh^ (Table 1, entries 7–12). Since **4** ^I‐BArF4^ and **4** ^H‐BArF4^ feature the exact same backbone structure as **4** ^Ch‐Z^, and because precursors **5** ^Ch^ should provide stronger Lewis basic chalcogen centers than **4** ^Ch‐Z^, it is very unlikely that the catalysts reported below act through activation modes other than chalcogen bonding (like π‐activation^17^ or hydrogen bonding).^18^

Next, catalyst **4** ^Te‐BArF4^ was applied in the benchmark reaction, as it was assumed that this compound should be the most powerful one in the family of compounds studied herein (Figure 1, **4** ^Ch‐Z^): in presence of 10 mol % of **4** ^Te‐BArF4^, >95 % yield of compound **3** was obtained after 4 h (Table 2, entry 1). To elucidate the role of the chalcogen center, and to compare the activity of catalysts based on lighter elements, **4** ^Se‐BArF4^ and **4** ^S‐BArF4^ were synthesized by simple anion exchange with TMABAr^F^_4_ from their known5c BF_4_‐derivatives (Scheme 2).

**Table 2:** Performance of the catalyst candidates in the reaction between indole **1** and carbonyl compound **2**.

Entry	Catalyst	Loading [mol %]	Yield of **3** [%]^[b,c,d,e]^	*k*_rel_^[f]^
1	**4** ^Te‐BArF4^	10	>95 (>95)	1000
2	**4** ^Se‐BArF4^	10	32 (16)	150
3	**4** ^S‐BArF4^	10	<5	–
4	**4** ^Te‐BF4^	10	95 (46)	350
5	**4** ^Te‐OTf^	10	95 (57)	400
6	**4** ^Te‐NTf2^	10	5 (2)	15
7	**4** ^Se‐BF4^	10	<5	–
8	**4** ^Se‐OTf^	10	<5	–
9	TMABAr^F^_4_	20^[a]^	<5	–
10	NEt_4_OTf	20^[a]^	<5	–
11	NMe_4_BF_4_	20^[a]^	<5	–
12	**4** ^Te‐BArF4^	7.5	>95 (82)	750
13	**4** ^Te‐BArF4^	5	61 (14)	75
14	**4** ^Te‐BArF4^	2.5	<5	–
15	**4** ^Se‐BArF4^	5	<5	–

[a] 20 mol % catalyst were used to ensure the same number of potential Lewis acidic centers as in bidentate catalysts. [b] ^1^H NMR yields of compound **3** after 24 h reaction time (with TES as internal standard). [c] In brackets the yields of compound **3** after 4 h reaction time are given. [d] Averaged values of a least two measurements. [e] No indication of catalyst decomposition was observed in any case [f] Relative reaction rates compared to halogen bond donor **4** ^I‐BArF4^ (see Supporting Information).

**Scheme 2:**
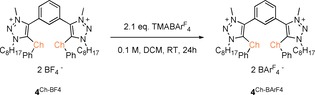
Anion exchange for **4** ^Se‐BF4^ and **4** ^S‐BF4^ to yield their corresponding BAr^F^_4_‐salts **4** ^Se‐BArF4^ and **4** ^S‐BArF4^. Ch = S and Se.

In the presence of 10 mol % of catalyst **4** ^Se‐BArF4^, 32 % of compound **3** was obtained after 24 h, but no reaction occurred with catalyst **4** ^S‐BArF4^ (Table 2, entries 2 and 3). These observations are in good agreement with the expected activity of chalcogen bond donors (S<Se<Te), which was also confirmed in earlier works.5c, 13 The same trend has been observed for halogen bonding catalysis in a Michael addition reaction, with iodinated compounds again being the most potent ones.15a

To elaborate the effect of the counter anions in this carbonyl activation with dicationic chalcogen bond donors, several other tellurium‐ and selenium‐based catalysts **4** ^Ch‐Z^ (Figure 1) were also tested. The hypothesis was that their catalytic activity should follow the inverse trend given by the coordinating ability of the respective anions (NTf_2_^−^>OTf^−^≈BF_4_^−^>BAr^F^_4_^−^), as the Lewis acidic chalcogen centers should then become increasingly accessible to substrates. And indeed, the expected order in catalytic performance was experimentally observed for the tellurium‐based catalysts: after 4 h of reaction time, **4** ^Te‐BF4^ and **4** ^Te‐OTf^ still produced 46 and 57 % yield, respectively, of product **3** (Table 2, entries 4 and 5), compared to the >95 % obtained with **4** ^Te‐BArF4^ (see above; after 24 h, both catalysts **4** ^Te‐BF4^ and **4** ^Te‐OTf^ also generated compound **3** in 95 % yield). The corresponding NTf_2_ salt was virtually inactive (with 5 % yield after 24 h; Table 2, entry 6), which was a surprisingly bad performance that was nevertheless in line with similar observations in our earlier study on a nitro‐Michael reaction.5c In contrast to the organotellurium compounds, the OTf or the BF_4_ derivatives of selenium‐based catalysts **4** ^Se‐Z^ did not show any further activity (Table 2, entries 7 and 8).

To exclude any catalytic effects based on the interactions of the anions with the substrate, TMABAr^F^_4_, NEt_4_OTf and NMe_4_BF_4_ were subsequently also tested in the reaction but proved to be inactive (Table 2, entries 9–11).

Given the strong activity of our best catalyst **4** ^Te‐BArF4^, we then investigated to which extent the catalyst load could be reduced while still satisfactory yields of the product could be obtained. To this end, the catalyst amount was reduced to 7.5, 5 and 2.5 mol %. Although the outcome of the run with 7.5 mol % of **4** ^Te‐BArF4^ was still comparable to our original results (>95 % yield after 24 h, Table 2, entry 12), the yield dropped markedly with a catalyst loading of 5 mol % (61 % after the same time, Table 2, entry 13). A further reduction in the amount of catalyst **4** ^Te‐BArF4^ to 2.5 mol % lead to no product formation (Table 2, entry 14), and the same was found with a reduction of the catalyst loading of **4** ^Se‐BArF4^ to 5 mol % (Table 2, entry 15).

Next, we determined relative rate accelerations induced by various catalysts based on an analysis of the initial reaction rates in the first 2 h of reaction time (Table 2; for a kinetic plot of selected catalysts, see Figure 2). The corresponding reaction rates *k*_rel_ are based on **4** ^I‐BArF4^ as a reference compound (*k*_rel_=1). The strongest catalyst **4** ^Te‐BArF4^ accelerates the reaction by about a factor of 1000, whereas the corresponding BF_4_ and OTf salts still provide accelerations by about 350–400 (Table 2, entries 1, 4 and 5). A reduction of the catalyst loading of **4** ^Te‐BArF4^ from 10 to 5 mol % decreased the relative reaction rate by a factor of about 14 (*k*_rel_=75, Table 2, entry 13). The analogue selenium derivative **4** ^Se‐BArF4^ induces an approximately 150‐fold faster reaction compared to the halogen bond donor (Table 2, entry 2), which is about 7‐fold lower than the tellurium compound. These comparisons once again clearly illustrate the superiority of tellurium‐based chalcogen‐bond donors compared to their selenium counterparts as well as the impact of non‐coordinating counterions for the activation of neutral compounds like carbonyl derivatives.

**Figure 2:**
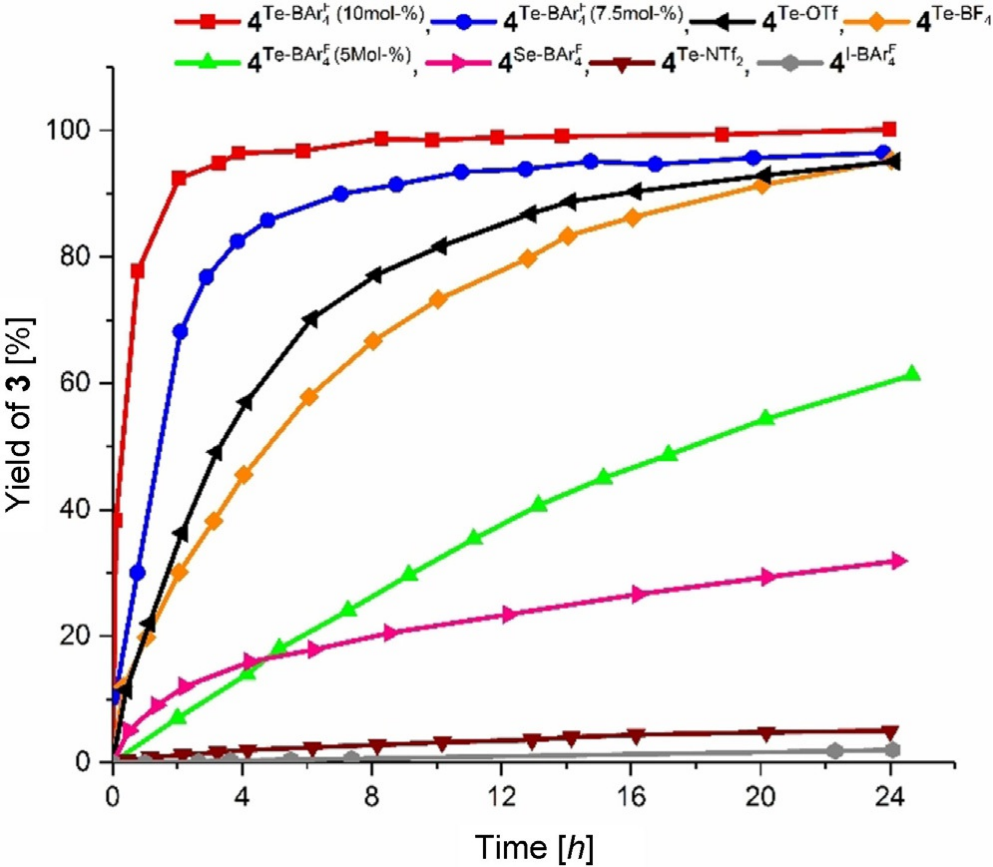
Kinetic plot for the reaction of indole **1** with *trans*‐crotonophenone (**2**) as yield versus time profile. The yields were determined by ^1^H NMR spectroscopy.

Subsequently, ^1^H NMR titration studies were performed to determine the binding strength^19^ of selected chalcogen bond donors with *trans*‐crotonophenone (**2**) and to then check whether there is a correlation between the catalytic activity of these compounds and their coordination strength to the substrate (Table 3). Surprisingly, all tested catalysts resulted in more or less the same binding constant (K ≈2 m ^−1^), independently of the chalcogen moiety or the counterion. These low binding constants are in line with published data for the coordination of a somewhat related halogen bond donor to cyclohexanone. For this case, a binding constant of 4 m ^−1^ was obtained,^20^ which is equal to the one of the strongest‐binding catalyst **4** ^Te‐BF4^ (Table 3, entry 1). Likely, though, all deviations in Table 3 are still within the margin of error of the titrations. Particularly puzzling is a comparison of the binding constants of **4** ^Te‐BArF4^, **4** ^Se‐BArF4^ and **4** ^S‐BArF4^ to *trans*‐crotonophenone (Table 3, entries 2–4), as the catalysts differ wildly in activity despite their similar binding. Obviously, one would have expected that a difference in Lewis acidity would also be reflected in the coordination data. It is possible, though, that the binding of the catalysts to the neutral substrate is so weak that any differences are evened out by other effects like further weak interactions or solvation effects. Surely, the decisive interaction of the catalysts is with the transition state of the reaction, in which the carbonyl oxygen will be somewhat negatively charged. It is plausible that the differences in Lewis acidity will manifest themselves more pronouncedly once the binding itself becomes reasonably strong. The data could also indicate that the reaction mechanism is more complex than a simple activation of the ketone by the chalcogen bond donor. In this regard, we note that orientating visual kinetic analyses^21^ with “different excess” experiments have indicated that the catalysts act as second‐order components in this reaction (while both substrates are first‐order). Surely, further mechanistic studies and computational investigations are necessary, which are however outside the scope of this publication.

**Table 3:** ^1^H NMR titration data for the binding of selected catalysts (hosts) to *trans*‐crotonophenone (**2**; guest) in deuterated methylene chloride at 25 °C.

Entry^[a]^	Catalyst	Binding Constant [m ^−1^]
1	**4** ^Te‐BF4^	4.0
2	**4** ^Te‐BArF4^	1.9
3	**4** ^Se‐BArF4^	2.0
4	**4** ^S‐BArF4^	1.8
5	**4** ^Te‐OTf^	2.6

Finally, a substrate screening with the best catalyst **4** ^Te‐BArF4^ and its selenium analogue **4** ^Se‐BArF4^ was performed, in which various indole derivatives—electron‐rich/poor as well as sterically demanding ones—were employed (Figure 3). In all cases it was initially confirmed that no background reactivity is present, and in fact even after 48 h reaction time no conversion to compounds **3 a**–**g** was observed. Then, the performance of both catalysts was compared.

**Figure 3:**
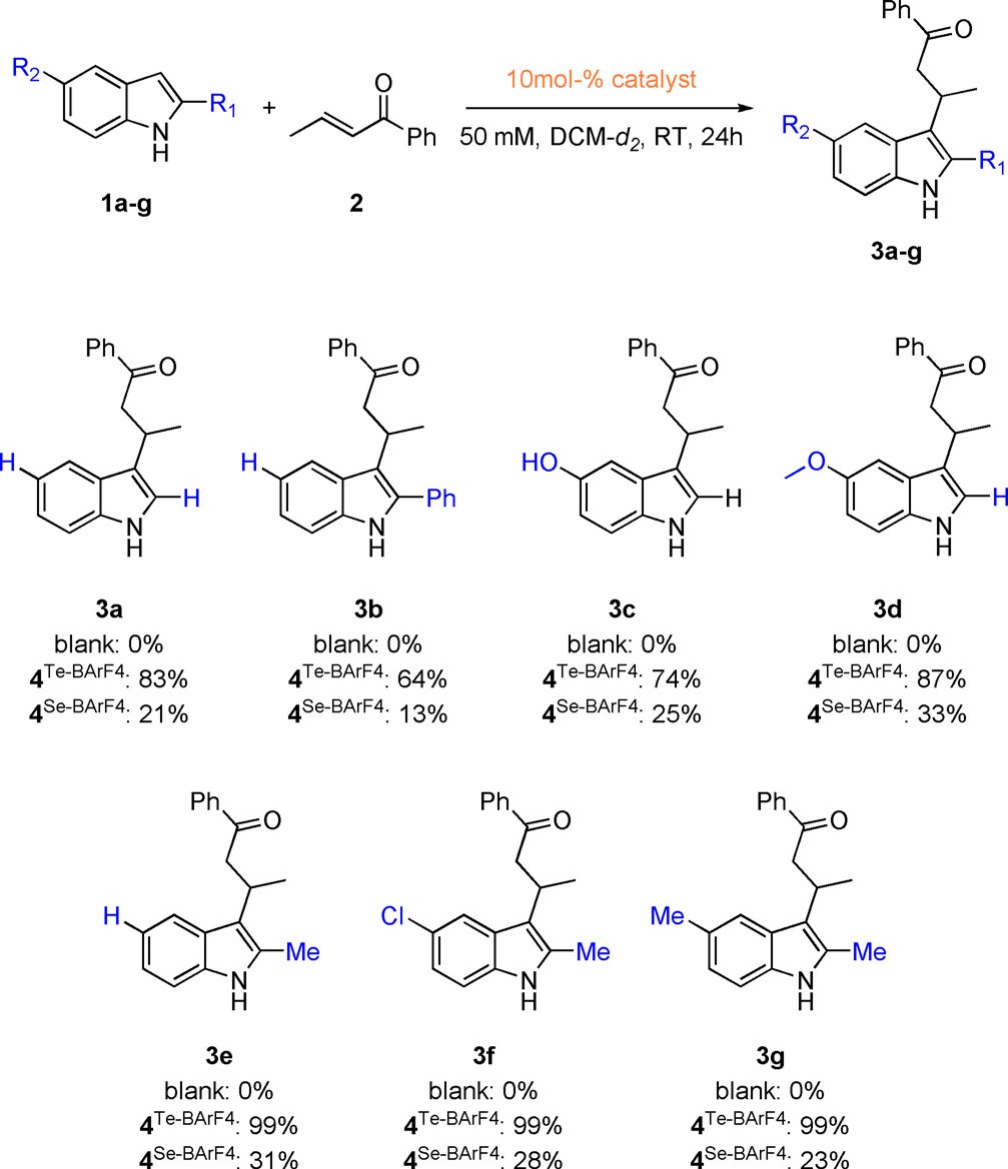
Substrate scope of the reaction of *trans*‐crotonophenone (**2**) with several indole derivatives (**1 a**–**g**). The yield for each compound in presence of the respective catalyst is given below the structure. In blue the different substituents R are highlighted. All reactions were run at least two times.

For compounds **3 a**–**d**, yields between 64–87 % were observed after 24 h when catalyst **4** ^Te‐BArF4^ was applied, whereas with **4** ^Se‐BArF4^ only 13–33 % conversion to compounds **3 a**–**d** was observed after the same time. By comparison, after 24 h reaction time and in presence of catalyst **4** ^Te‐BArF4^, compounds **3 e**–**g** were quantitatively converted. With the selenium analogue **4** ^Se‐BArF4^, only 23–31 % yield of compounds **3 e**–**g** was achieved after the same time. Overall, substituents at position R^1^—even methyl groups—seem to lead to slower conversions, whereas the nucleophilicity of the indole derivative^22^ does not seem to be a decisive factor, as more nucleophilic derivatives (compare **1 d** vs. **1 f**) were converted slower in some cases. Similar trends were observed with the selenium‐based catalyst **4** ^Se‐BArF4^ (compare **1 d** vs. **1 g**), even though the yields range only from 13–33 % after 24 h.

In conclusion, the first activation of carbonyl compounds by tellurium‐based chalcogen bond donors was presented. This is also only the second such activation by chalcogen bonding in general. The prototypical Michael addition reaction investigated in this paper can be accelerated by a factor of up to 1000 with bis(triazolium)benzene‐based chalcogen bond donors in comparison to their virtually inactive iodinated halogen bonding analogues. Even with a catalyst load of only 7.5 mol % of the strongest chalcogen bond donor, quantitative conversion to product was found after 24 h. The activity of the dicationic catalysts was strongly dependent on their counterion, with non‐coordinating ones like BAr^F^_4_ expectedly providing the most active compounds.

Future work will deal with detailed mechanistic studies on this and a related nitro‐Michael reaction, as the activity of the catalyst was found to be unrelated to their binding strength to the carbonyl substrate. In addition, the application of the presented catalysts will be extended towards other types of reactions and other substrate classes, and the catalyst structures will be further optimized by preorganization.^23^

## Conflict of interest

The authors declare no conflict of interest.

## Supporting information

As a service to our authors and readers, this journal provides supporting information supplied by the authors. Such materials are peer reviewed and may be re‐organized for online delivery, but are not copy‐edited or typeset. Technical support issues arising from supporting information (other than missing files) should be addressed to the authors.

Supplementary
