# Supplementary material for: Carbonyl Activation by Selenium‐ and Tellurium‐Based Chalcogen Bonding in a Michael Addition Reaction
Source: Chemistry. 2020 Jan 21;26(6):1258–62. doi: 10.1002/chem.201905057 (PMC7027547; doi:10.1002/chem.201905057)
Supplement: Supplementary file 1 — Supplementary [file CHEM-26-1258-s001.pdf]

CHEMISTRY
A European Journal

Supporting Information

Carbonyl Activation by Selenium- and Tellurium-Based Chalcogen Bonding in a Michael Addition Reaction

Patrick Wonner, Tim Steinke, Lukas Vogel, and Stefan M. Huber*[a]

chem_201905057_sm_miscellaneous_information.pdf

Table of Contents

1. Experimental Section ... 2

1.1. Experimental Conditions ... 2

1.2. Solvents ... 2

1.3. Chemicals ... 2

1.4. Analysis Methods ... 2

1.5. Synthesis of Known Compounds ... 3

1.6. Synthesis of New Compounds ... 9

1.7. ^{1}H NMR Reaction Setup ... 14

2. Determination of k_{rel} values ... 16

3. ^{1}H NMR Titration Experiments ... 17

4. Literature ... 21

1. Experimental Section

1.1. Experimental Conditions

All experiments were carried out in flame dried *Schlenk* flasks under argon atmosphere and with dry solvents. Solvents used for chromatography were previously distilled. All used chemicals are commercially available and were used without further purification. Thin-layer chromatography was performed by using *Merck TLC aluminium sheets* (silica gel 60, F254). Column chromatography was performed with silica gel (grain size 0.04-0.063 cm, *Merck Si60*) at atmospheric pressure (1-1.5 atm using in some cases a hand pump). The corresponding solvents that were used as eluents as well as the R_f values are listed at the corresponding experiment. Detection of the substances was obtained by fluorescence detection under UV light (wavelength λ = 254 nm).

1.2. Solvents

Dry DCM, ether and THF were received from a *MBRAUN MB SPS-800*. At first solvents were distilled, dried over 4 Å molecular sieve and finally dried on an Alox column. Further dry solvents were dried over flame dried 4Å molecular sieve. The moisture content was determined with a Karl Fischer *Titroline®7500KF trace*.

1.3. Chemicals

Chemicals were obtained from *ABCR*, *Alfa Aesar*, *Carbolution*, *Merck*, *ChemPur*, *Sigma Aldrich* or *VWR*. Commercially available reagents and starting materials were used without further purification (unless mentioned otherwise).

1.4. Analysis Methods

1.4.1. NMR Spectroscopy

^{1}H NMR spectra and ^{13}C NMR spectra were recorded with an *Aviii 300* and a *Bruker DRX 400* spectrometer at 298.5 K. ^{19}F NMR spectra were recorded with a *Bruker DPX-250 NMR* spectrometer at 298.5 K. Peaks were referenced to residual ^{1}H signals and ^{13}C signals from the deuterated solvents and are reported in parts per million (ppm). For ^{1}H NMR spectroscopically data, ^{13}C NMR spectroscopically data and ^{19}F NMR spectroscopically data, multiplicity, the relative integral and the coupling constant (*J* in Hz) are indicated if possible.

1.4.2. *ATR-IR Measurements*

IR spectra were recorded with a *Shimadzu IR Affinity - 1S* spectrometer and are reported in ν = cm^{-1} and are indicted with w (weak), m (middle), s (strong) or vs (very strong).

1.4.3. *EI, ESI and MALDI-TOF Measurements*

Mass spectra were recorded with either a Bruker Daltonics Esquire 6000 instrument (ESI), a VG Instruments Autospec / EBEE-Geometrie (EI) or a *Ultraflex III* mass spectrometer from *Bruker* (MALDI-TOF).

1.4.4. *Elemental Analysis*

CHNS Elemental Analysis was performed with a *vario Micro cube* from *Elementar Analysentechnik*.

1.4.5. *Balance for Stock Solutions*

Starting materials for stock solutions were weight in on a *Mettler Toledo XSR 105 Dual Range* balance.

1.5. *Synthesis of Known Compounds*

1.5.1. *Compound $\mathbf{4}^{H\text{-}BArF4}$*

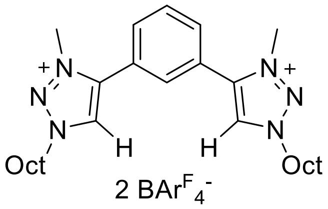

Compound **4**$^{H\text{-}BArF4}$ was synthesized according to literature.[1]

1.5.2. *Compound $\mathbf{4}^{I\text{-}BArF4}$*

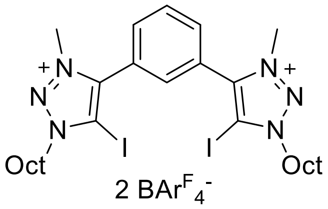

Compound **4**$^{I\text{-}BArF4}$ was synthesized according to literature.[1b]

1.5.3. Compound TMABAr^{F}$_{4}$

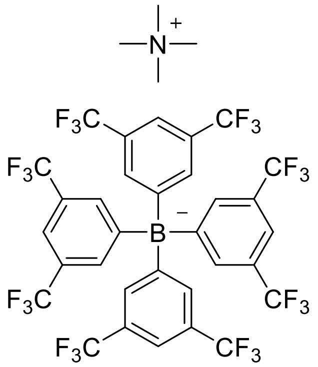

Compound **TMABAr^{F}$_{4}$** was synthesized according to literature.[2]

1.5.4. Synthesis of 5^{S}

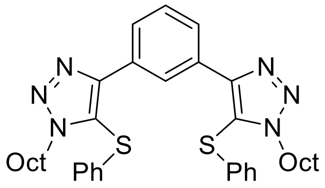

Compound **5^{S}** was synthesized according to literature.[3]

1.5.5. Synthesis of 5^{Se}

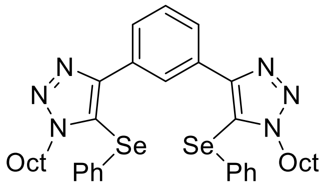

Compound **5^{Se}** was synthesized according to literature.[3]

1.5.6. Synthesis of 5^{Te}

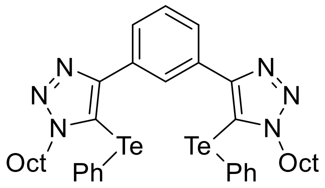

Compound **5^{Te}** was synthesized according to literature.[3]

1.5.7. *Synthesis of $4^{Se\text{-}BF4}$*

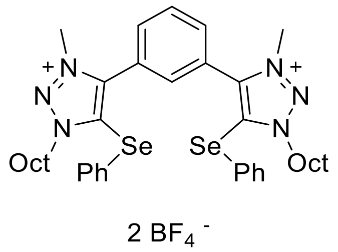

Compound **4**$^{\mathbf{Se\text{-}BF4}}$ was synthesized according to literature.[3]

1.5.8. *Synthesis of $4^{Te\text{-}BF4}$*

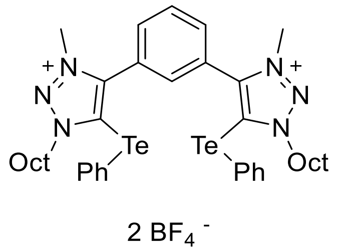

Compound **4**$^{\mathbf{Te\text{-}BF4}}$ was synthesized according to literature.[3]

1.5.9. *Synthesis of $4^{Te\text{-}OTf}$*

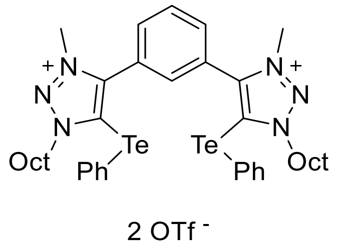

Compound **4**$^{\mathbf{Te\text{-}OTf}}$ was synthesized according to literature.[3]

1.5.10. *Synthesis of $4^{Te\text{-}NTf2}$*

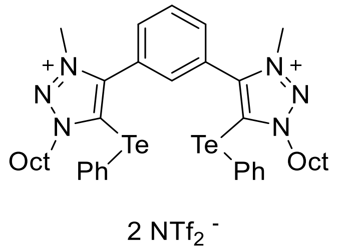

Compound **4**$^{\mathbf{Te\text{-}NTf2}}$ was synthesized according to literature.[3]

1.5.11. Synthesis of $4^{Te\text{-}BArF4}$

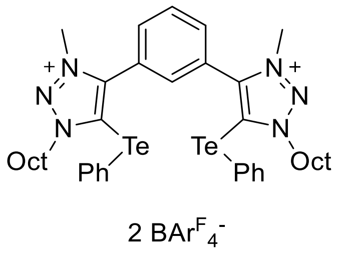

Compound $\mathbf{4}^{\mathbf{Te\text{-}BArF}}$ was synthesized according to literature.[3]

1.5.12. Compound 3a

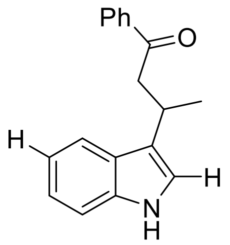

3a

Spectroscopical data for compound **3a** are according to literature.[4]

1.5.13. Compound 3b

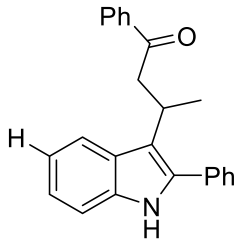

3b

Spectroscopical data for compound **3b** are according to literature.[5]

1.5.14. Compound 3c

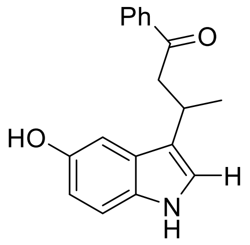

3c

Spectroscopical data for compound **3c** are according to literature.[6]

1.5.15. Compound 3d

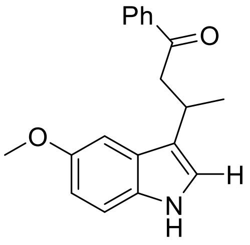

3d

Spectroscopical data for compound **3d** are according to literature.[7]

1.5.16. Compound 3e

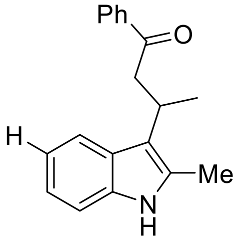

3e

Spectroscopical data for compound **3a** are according to literature.[8]

1.5.17. Compound 3f

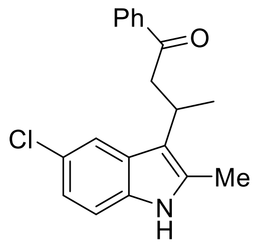

3f

Spectroscopical data for compound **3a** are according to literature.[7]

1.5.18. Compound 3g

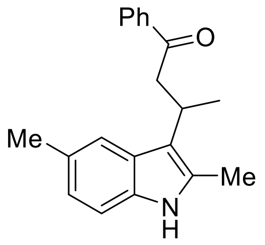

3g

Spectroscopical data for compound **3a** are according to literature.[7]

1.6. *Synthesis of New Compounds*

1.6.1. *Synthesis of compound 4^S-BArF4^*

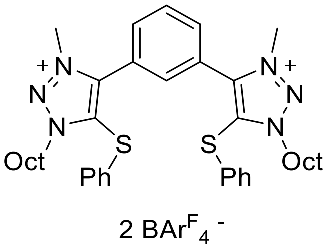

Compound **4**$^{\mathbf{S\text{-}BArF4}}$ was synthesized after the following procedure[3]:300 mg (0.35 mmol, 1 eq.) of compound **4**$^{\mathbf{S\text{-}BF4}}$ were dissolved in 35 ml dry chloroform (0.01 M) and 820 mg TMABAr$^F{}_4$ (0.88 mmol, 2.5 eq.) were added to the solution, which was then stirred for 18 h at room temperature. Subsequently, the solvent was removed and the solid was taken up in ether and the solution was cooled to -78°C until a precipitate (TMABF$_4$) was formed, which was filtered off and was washed two times with -78 °C cold ether. Then, the solvent of the organic solution was removed, and the solid residue was diluted with a little amount of chloroform and cooled to - 55 °C until a solid precipitated (excess of TMABAr$^F{}_4$). The mixture was filtered, and the solid residue was washed three times with -55 °C cold chloroform. After the solvent of the organic phase was removed, **4**$^{\mathbf{S\text{-}BArF4}}$ was obtained with 600 mg (0.25 mmol, 71%)yield as yellowish sticky resin.

^{1}H NMR (300 MHz, Chloroform-*d*):

δ [ppm] = 7.75 (s, 2H, C$H_{arom.}$), 7.70 (s, 16H, C$H_{arom.}$), 7.46 (s, 10H, C$H_{arom.}$), 7.15 (t, J = 7.52 Hz, 2H, C$H_{arom.}$), 7.10 (t, J = 7.56 Hz, 4H, C$H_{arom.}$), 6.98 (d, J = 8.74 Hz, 4H, C$H_{arom.}$), 4.46 (t, J = 7.35 Hz, 4H, CH_2), 3.98 (s, 6H, CH_3), 1.80 (m, 4H, CH_2), 1.24 (m, 20H, CH_2), 0.80 (t, J = 6.47 Hz, 6H, CH_3).

^{13}C NMR (75 MHz, Chloroform-*d*):

δ [ppm] = 160 (m, $C_{\text{arom.}}$), 143.11 ($C_{\text{arom.}}$), 135.86 ($C_{\text{arom.}}$), 134.46 ($C_{\text{arom.}}$), 132.98 ($C_{\text{arom.}}$), 130.29 (d, J = 11.9 Hz, $C_{\text{arom.}}$), 128.61 (d, J = 31.1 Hz, $C_{\text{arom.}}$), 126.64 ($C_{\text{arom.}}$), 125.99 ($C_{\text{arom.}}$), 122.65 ($C_{\text{arom.}}$), 122.38 ($C_{\text{arom.}}$), 117.24 ($C_{\text{arom.}}$), 52.94 (CH$_2$), 38.85 (CH$_3$), 31.17 (CH$_2$), 28.33 (d, J = 13.4 Hz, CH$_2$), 25.77 (CH$_2$), 22.15 (CH$_2$), 13.58(CH$_3$).

^{19}F NMR (235 MHz, Chloroform-*d*):

δ [ppm] = -62.45 (s, C*F*$_3$)

ATR-IR:

$\tilde{\nu}$ [cm^{-1}] = 2933 (w), 2864 (w), 1610 (m), 1444 (w), 1352 (vs), 1271 (vs), 1111 (vs), 1001 (w), 931 (m), 885 (s), 839 (s), 744 (m), 711 (vs), 680 (vs), 669 (vs), 619 (w), 580 (w), 449 (m).

ESI-MS:

m/*z (+)* = calc. 341.19 [M^{2+}], found 770.82 [M+BAr$^F{}_4{}^-$]$^{2+}$.

m/*z (-)* = calc. 863.06 [M$^-$], found 862.70 [M$^-$].

CHNS:

	C	H	N	S
calc.	51.84	3.26	3.49	2.66
found	51.29	3.46	2.89	2.07

1.6.2. Synthesis of compound 4$^{Se\text{-}BArF4}$

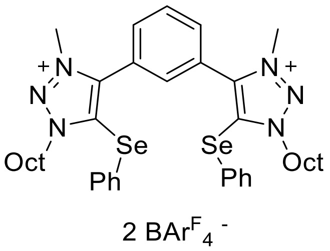

Compound **4$^{Se\text{-}BArF4}$** was synthesized after the following procedure[3]:300 mg (0.32 mmol, 1 eq.) of compound **4$^{Se\text{-}BF4}$** were dissolved in 32 ml dry chloroform (0.01 M) and 650 mg TMABAr$^F{}_4$ (0.69 mmol, 2.2 eq.) were added to the solution, which was then stirred for 18 h at room temperature. Subsequently, the solvent was removed and the solid was taken up in ether and the solution was cooled to -78°C until a precipitate (TMABF$_4$) was formed which was filtered off and was washed two times with -78 °C cold ether. Then, the solvent of the organic solution was removed, and the solid residue was diluted with a little amount of chloroform and cooled to - 55 °C until a solid precipitated (excess of TMABAr$^F{}_4$). The mixture was filtered, and the solid residue was washed three times with -55 °C cold chloroform. After the solvent of the organic phase was removed, **4$^{Se\text{-}BArF4}$** was obtained with 540 mg (0.21 mmol, 68%) yield as yellowish sticky resin.

^{1}H NMR (300 MHz, Chloroform-*d*):

δ [ppm] = 7.99 (d, *J* = 3.8 Hz, 1H, C*H*$_{arom.}$), 7.90 (s, 1H, C*H*$_{arom.}$), 7.82 (s, 1H, C*H*$_{arom.}$), 7.70 (m, 14H, C*H*$_{arom.}$), 7.47 (d, *J* = 5.8 Hz, 9H, C*H*$_{arom.}$), 7.24 (m, 4H, C*H*$_{arom.}$), 7.15 (d, *J* = 4.3 Hz, 8H, C*H*$_{arom.}$), 4.54 (dd, *J* = 8.6, 6.7 Hz, 4H, C*H*$_2$), 4.06 (d, *J* = 1.2 Hz, 6H, C$_3$), 1.88 (m, 4H, C*H*$_2$), 1.35 (m, 20H, C*H*$_2$), 0.85 (m, 6H, C*H*$_3$).

^{13}C NMR (75 MHz, Chloroform-*d*):

δ [ppm] = 162.45 (d, *J* = 49.9 Hz, *C*$_{arom.}$), 145.01 (*C*$_{arom.}$), 134.91(*C*$_{arom.}$), 132.83 (*C*$_{arom.}$), 130.85 (*C*$_{arom.}$), 130.38 (*C*$_{arom.}$), 129.04 (d, *J* = 31.9 Hz, *C*$_{arom.}$), 126.44 (*C*$_{arom.}$), 124.71 (*C*$_{arom.}$), 123.81 (*C*$_{arom.}$), 122.82 (*C*$_{arom.}$), 117.66 (*C*$_{arom.}$), 68.13 (*C*H$_2$), 54.10 (*C*H$_2$), 39.20 (*C*H$_3$), 31.69 (*C*H$_2$), 29.22 (*C*H$_2$), 28.85 (d, *J* = 10.4 Hz, *C*H$_2$), 26.26 (*C*H$_2$), 25.62 (*C*H$_2$), 22.63 (*C*H$_2$), 14.08 (*C*H$_3$).

^{19}F NMR (235 MHz, Chloroform-*d*):

δ [ppm] = -62.43 (s, C*F*$_3$).

ATR-IR:

$\tilde{\nu}$ [cm^{-1}] = 2931 (w), 2862 (w), 1610 (m), 1469 (w), 1442 (w), 1352 (vs), 1271 (vs), 1111 (vs), 1020 (w), 999 (w), 931 (w), 885 (s), 839 (s), 742 (s), 711 (vs), 680 (vs), 669 (vs), 619 (w), 578 (w), 468 (w) 449 8m), 403 (w).

ESI-MS:

m/*z (+)* = calc. 389.13 [M^{2+}], found 813.04 [M+BAr$^{F}_{4}$+Li]$^{2+}$.

m/*z (-)* = calc. 863.06 [M^{-}], found 862.71 [M^{-}].

CHNS:

	C	H	N	S
calc.	49.90	3.14	3.36	/
found	50.24	3.16	3.55	/

1.6.3. Synthesis of compound $4^{Se\text{-}OTf}$

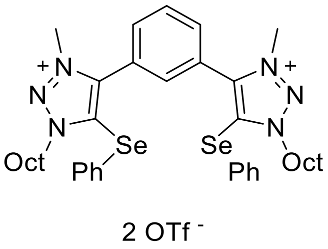

Compound **$4^{Se\text{-}OTf}$** was synthesized from **5^{Se}**. 508 mg of Compound **5^{Se}** (0.68 mmol, 1 eq.) were added to a flame dried Schlenk flask and were dissolved in 68 ml dry DCM (0.01 M). Subsequently, 0.22 ml methyl triflate (0.21 mmol, 3 eq.) were added and the mixture was stirred for 24 h. Removal of the solvent yielded a crude residue which was dissolved in a little amount of DCM and precipitated through addition of ether as sticky resin. Finally, the sticky resin was washed with pentane to yield 351 mg **$4^{Se\text{-}OTf}$** (0.33 mmol, 48%) as solid.[3]

^{1}H NMR (300 MHz, Chloroform-*d*):

δ [ppm] = 8.23 (t, ^4J = 1.7 Hz, 1H,CH_{ar}), 7.91 (dd, ^3J = 7.8 Hz, ^4J = 1.8 Hz, 2H, CH_{ar}), 7.65 (t, J = 7.9 Hz, 1H, CH_{ar}), 7.24 (m, 10H, CH_{ar}), 4.57 (t, ^3J = 7.7 Hz, 4H, CH_{ar}), 4.22 (s, 6H, CH_3), 1.90 (p, ^3J = 7.5 Hz, 4H, CH_2), 1.28 (m, 20H, CH_2), 0.88 (t, ^3J = 6.8 Hz, 6H, CH_3).

^{13}C NMR (75 MHz, Chloroform-*d*):

δ [ppm] = 145.73 ($C_{arom.}$), 134.03 ($C_{arom.}$), 132.77 ($C_{arom.}$), 132.16 ($C_{arom.}$), 130.65 ($C_{arom.}$), 129.61 ($C_{arom.}$), 129.36 ($C_{arom.}$), 126.25 ($C_{arom.}$), 123.90 ($C_{arom.}$), 53.65 (CH$_2$), 39.49 (CH$_3$), 31.80 (CH$_2$), 28.98 (CH$_2$), 28.88 (CH$_2$), 26.38 (CH$_2$), 22.70 (CH$_2$), 14.20 (CH$_3$).

^{19}F NMR (235 MHz, Chloroform-*d*):

δ [ppm] = -78.38 (s, 6F, $^-$O3S-C*F*3).

ATR-IR:

$\tilde{\nu}$ [cm^{-1}] = 3057 (w), 2955 (w), 2924 (m), 2855 (w), 1576 (w), 1558 (w), 1541 (w), 1481 (m), 1468 (w), 1439 (w), 1339 (w), 1312 (w), 1259 (vs), 1223 (m), 1152 (s), 1103 (w), 1066 (w), 1028 (s), 912 (w), 846 (m), 799 (m), 737 (m), 691 (m), 665 (w), 633 (vs), 571 (m), 517 (m), 459 (m).

MALDI-TOF-MS:

m/*z* = calc. 927 [M-OTf]$^+$, 778 [M-2OTf]$^+$, 622 [M-2OTf-SePh]$^+$;

found 931 [M-OTf]$^+$, 774 [M-2OTf]$^+$, 623 [M-2OTf-SePh].

CHNS:

	C	H	N	S
calc.	46.93	5.06	7.82	5.96
found	46.09	4.92	7.74	5.11

1.7. ^{1}H NMR Reaction Setup

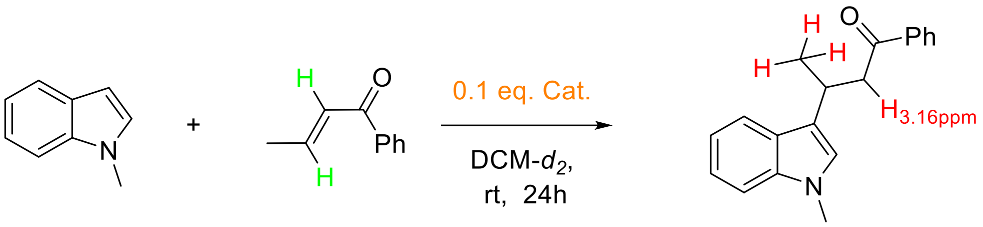

1 **2** **3**

Scheme 1: Chalcogen bond catalysed Michael addition reaction of 5-methoxyindole and trans-β-nitrostyrene in presence of **7^{TeBArF}**.

To a dry NMR tube, freshly prepared stock solutions of the respective catalyst (200 µL, 0.1 eq., mM), 1-methylindole (**1**) (200 µL, 1 eq., mM) and *trans*-β-crotonophenone (**2**) (200 µL, 1 eq., mM) in deuterated methylene chloride were added, sealed and the NMR tube was shaken for 1 min.. Afterwards, periodically ^{1}H NMR experiments (see Figure 1) were performed with a total duration of 24 h at room temperature.

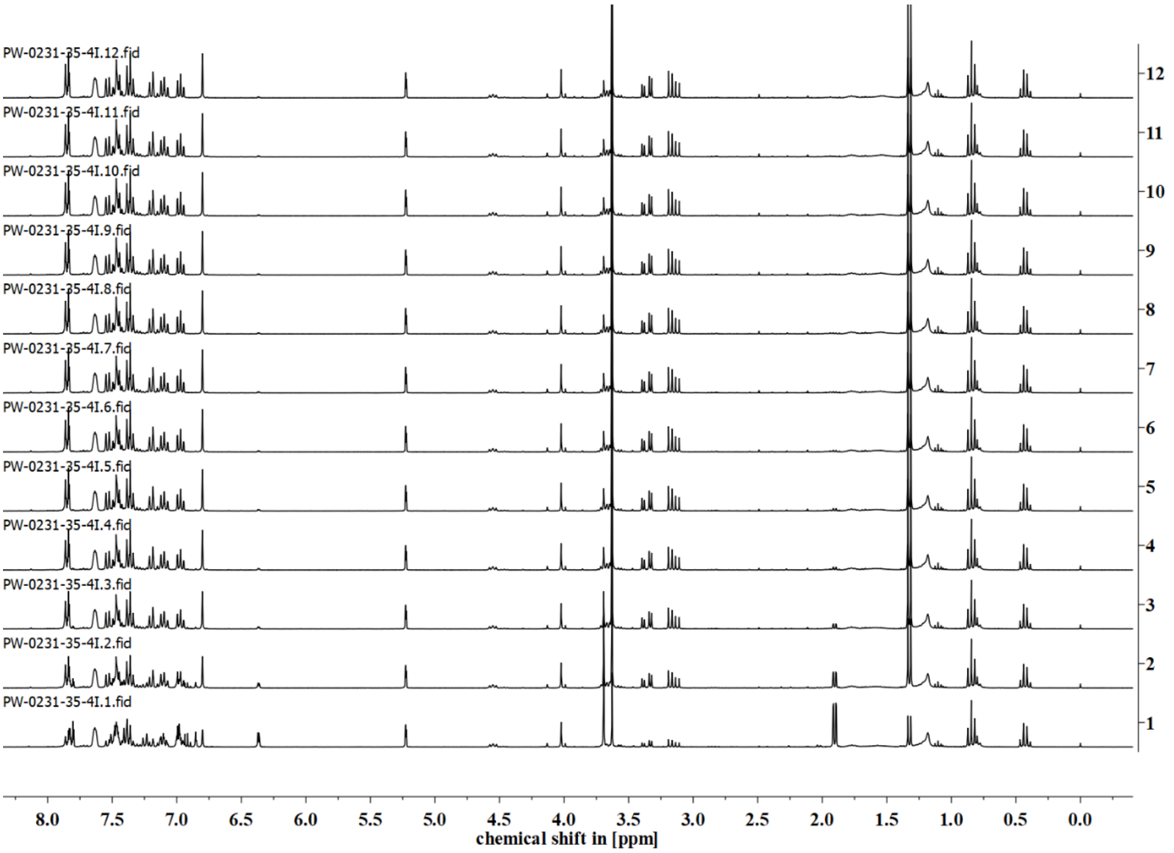

Figure 1: ^{1}*H NMR spectrum of the reaction between 1-methlyindole (***1***) and* trans-*β-crotonophenone (***2***) for the period of 24h (from bottom to top) in presence of* **4$^{Te\text{-}BArF4}$**. *Bottom spectrum after approximately 5 min. and the top spectrum after 24 h.*

As internal standard 0.125 eq. tetraethyl silane (TES) were added to the *trans*-β-crotonophenone (**2**) stock solution to check if there is decomposition of any compound as well as an integration standard for the determination of the yield of compound **3**. Hereto, the quartet of TES (0.51 ppm) was integrated as 1 and the integration of the characteristic signal at 3.16 pm gave directly the yield of product compound **3** (See Figure 2).

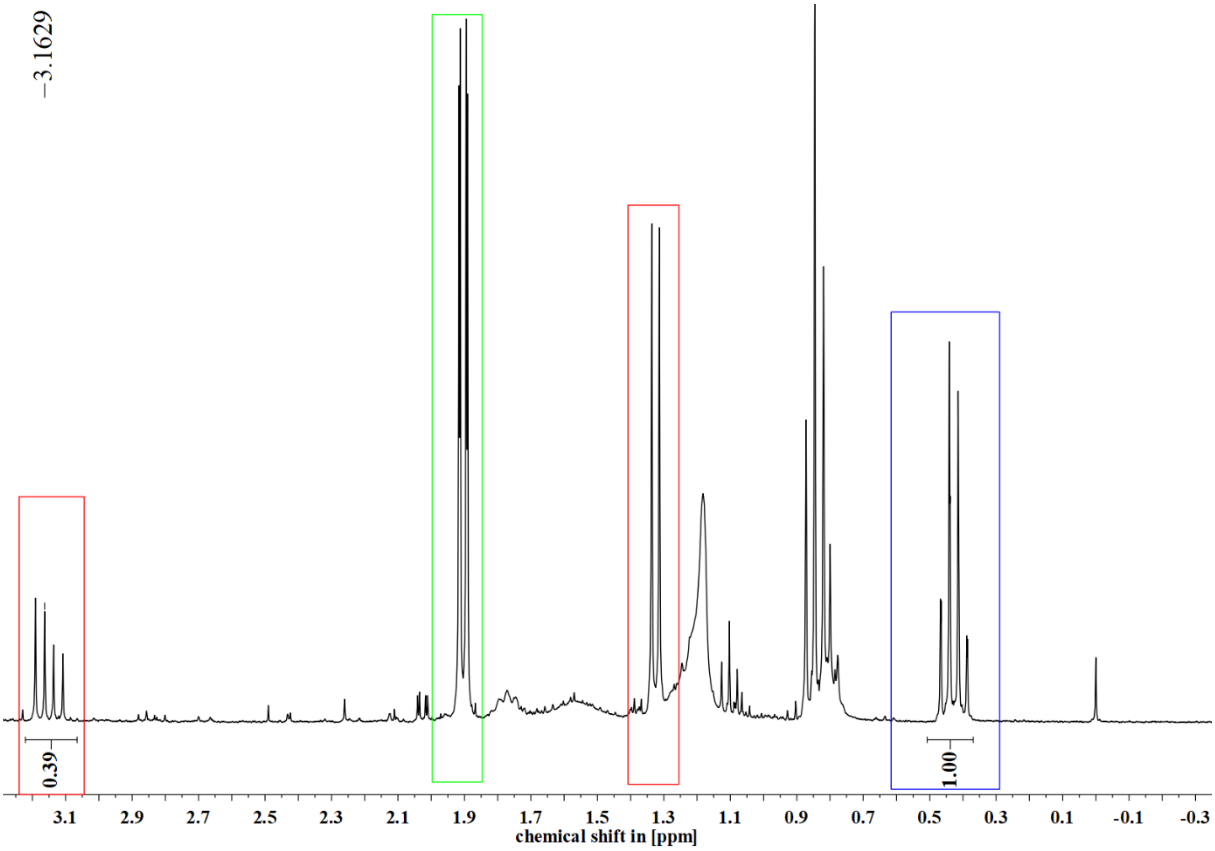

*Figure 2: ^{1}H NMR spectrum of the reaction between 1-methylindole (**1**) and trans-β-crotonophenone (**2**) in presence of 10 mol-% **7**$^{Te\text{-}BArF4}$ after 5 minutes. In red the signals of product **3**, in blue the TES standard. In this example 39% of compound **3** were obtained after 5 min. reaction time. At 3.16 ppm the typical signal of the proton of the product in α-position to the carbonyl is shown. The doublet in the red frame displays the methyl group in β-position of the carbonyl moiety and can be equally used to determine the NMR conversion. In green the methyl group of trans-β-crotonophenone (**2**) is shown.*

2. Determination of k_{rel} values

k_{rel} was determined by a linear fit from the kinetic plot of the reaction (see **Figure 3**). To this end, the gradient between zero hours and 2-2.6 h and the corresponding yield of **3** was determined for selected experiments. All used values for the slope determination are rounded to 2 h and the next highest number (**Table 1**). The reaction with halogen bond donor $\mathbf{4}^{\mathbf{I\text{-}BArF4}}$ was chosen as reference slope with a value of k_{rel} = 1. All other k_{rel} values were referred to this value (**Table 1**).

Table 1: Values for slope determination.

Entry	Catalyst	Mol-%	Time / h	~Yield of 3 / %	~Slope = $(\frac{\Delta y}{\Delta x})$	k_{rel}
1	$\mathbf{4}^{I\text{-}BArF4}$	10	2.65	0.12	0.045	1
2	$\mathbf{4}^{Te\text{-}BArF4}$	10	2	92	46	1000
3	$\mathbf{4}^{Te\text{-}BArF4}$	7.5	2	68	34	750
4	$\mathbf{4}^{Te\text{-}BArF4}$	5	2	7	3.5	75
5	$\mathbf{4}^{Te\text{-}BF4}$	10	2	30	15	350
6	$\mathbf{4}^{Te\text{-}OTf4}$	10	2	36	18	400
7	$\mathbf{4}^{Te\text{-}NTf}$	10	2.16	1.2	0.56	15
8	$\mathbf{4}^{Se\text{-}BArF4}$	10	2.21	12	5.4	150

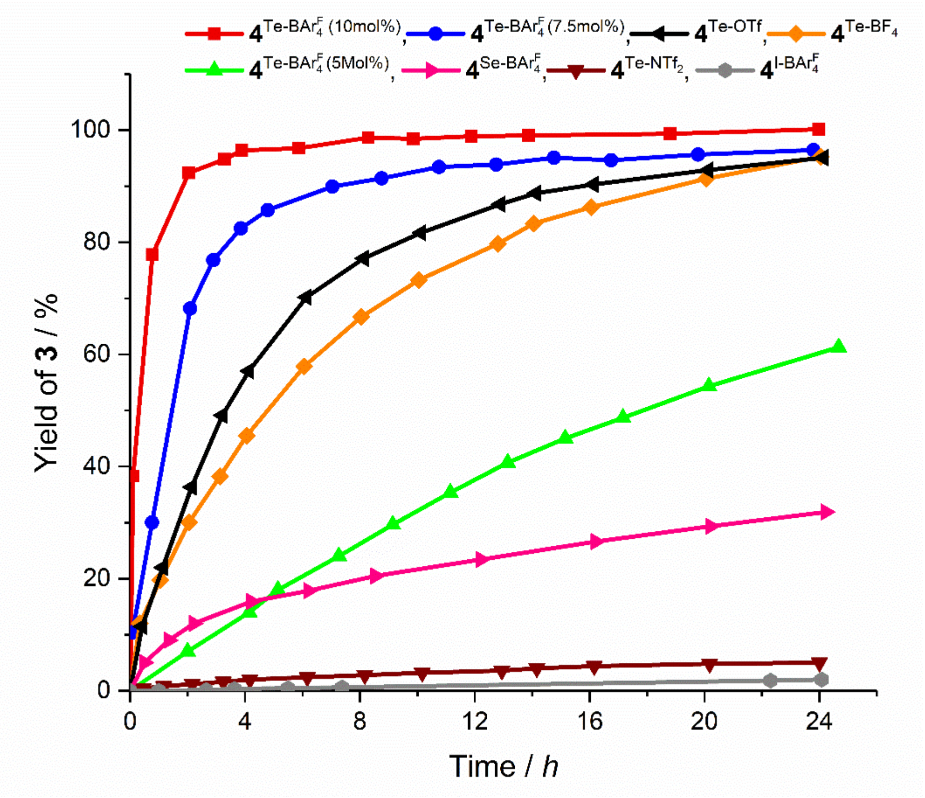

*Figure 3: Kinetic plot of the reaction between 1-methylindole (**1**) and trans-β-crotonophenone (**2**)*

3. ^{1}H NMR Titration Experiments

For pipetting, *Hamilton*®-syringes were used. All experiments were conducted at ambient temperature (298.5 K) and in *Wildmad*®*screw-cap* NMR tubes. 4 µmol of the host were dissolved in deuterated methylene chloride to give a 40 mM stock solution. A stock-solution of trans-β-crotonophenone (**2**) (ChB-acceptor / guest) was prepared as 4 M solution. For the titration 100 µl of the host were added to the NMR tube and diluted with 400 µl deuterated methylene chloride. Then the respective amount of guest solution (1 eq. ≡1 µl) was added for each data point as shown in Table 2. The ^{1}H NMR-spectra were measured with a *Bruker DRX 400*. ^{1}H NMR spectrum were measured with 16 scans and the host to guest ratio was checked by integration of the signals and corrected if necessary.

Table 2: ***Overview of Host Addition.***

**Equivalents**	**Added amount (µl) of the guest solution**
1	1
2	1
3	1
4	1
5	1
10	5
15	5
20	5
25	5
30	5
40	10
60	20
80	20
100	20
150	50
200	50

For the determination of the binding constants the shift of the N-*Me* protons or the ChPh protons from the respective catalyst were observed relative to the signal of the solvent. The measured shifts were plotted against the guest-equivalents and the resulting curve was fitted using http://supramolecular.org/.[9] For the calculations of the binding constants (K) a 1:1 binding was assumed.

In the following, titration plots for all tested compounds are given as well as a table with the binding constants.

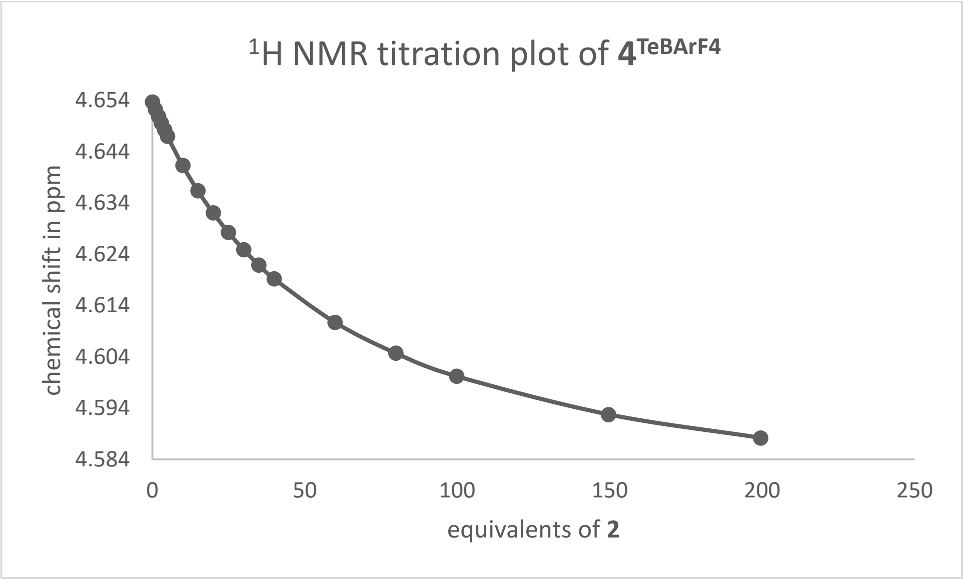

Figure 4 [1]*H NMR titration plot of* ***4***$^{\mathbf{TeBArF4}}$ *with trans-β-nitrostyrene (**2**) in* CD_2Cl_2.

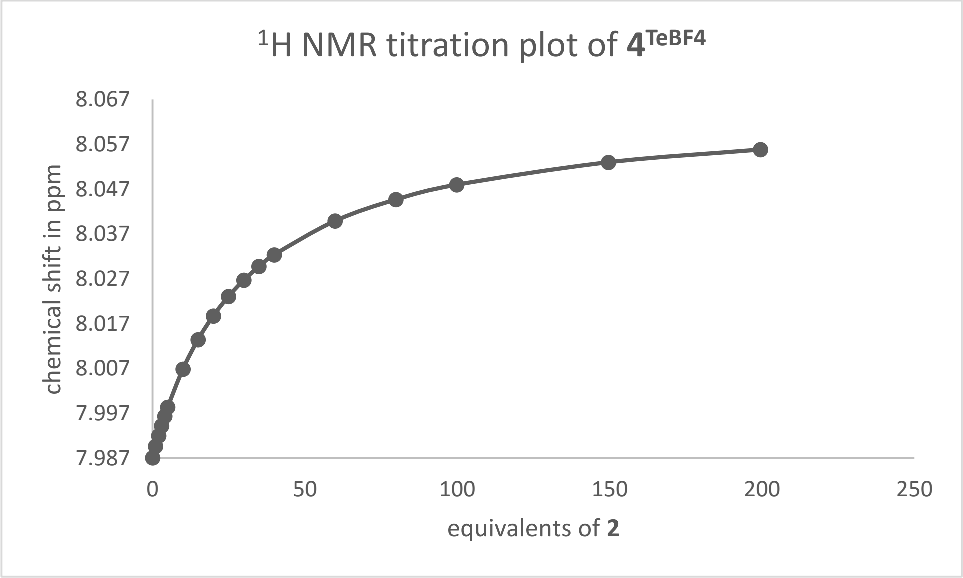

Figure 5: [1]*H NMR titration plot of* ***4***$^{\mathbf{TeBF4}}$*with trans-β-nitrostyrene (**2**) in* CD_2Cl_2.

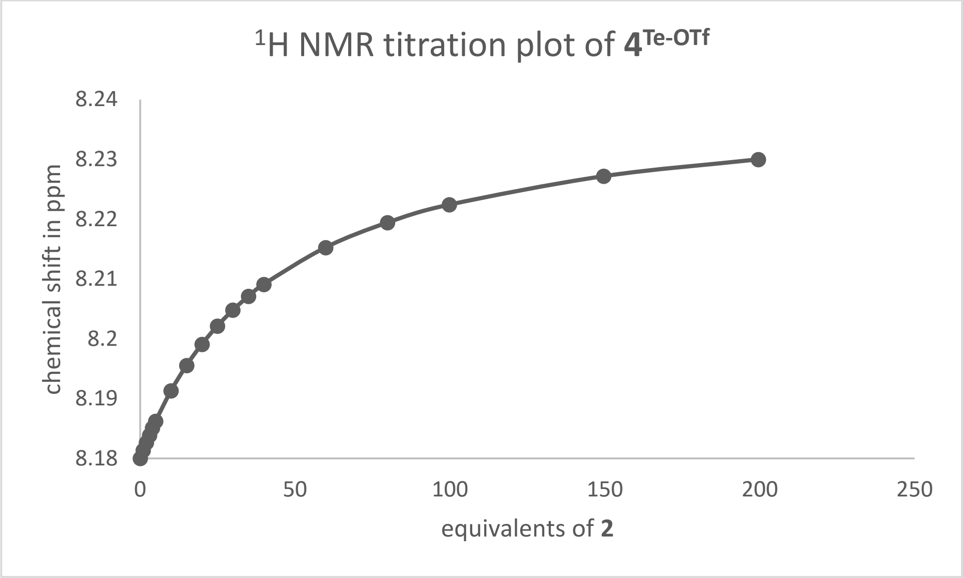

Figure 6: ^{1}*H NMR titration plot of* $\mathbf{4}^{\textit{\textbf{Te-OTf}}}$ *with trans-β-crotonophenone (**2**) in* CD_2Cl_2.

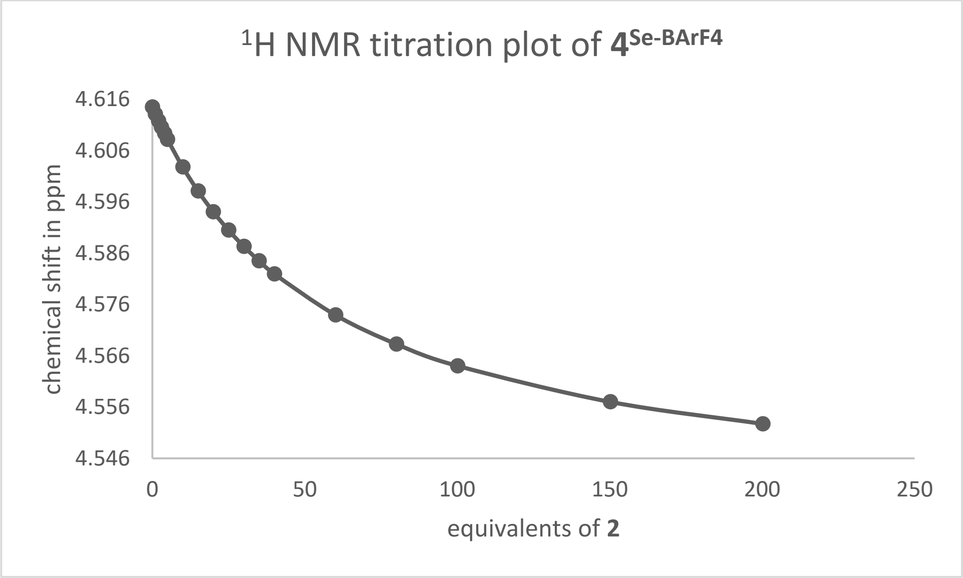

Figure 7: ^{1}*H NMR titration plot of* $\mathbf{4}^{\textit{\textbf{Se-BArF4}}}$ *with trans-β-crotonophenone (**2**) in* CD_2Cl_2.

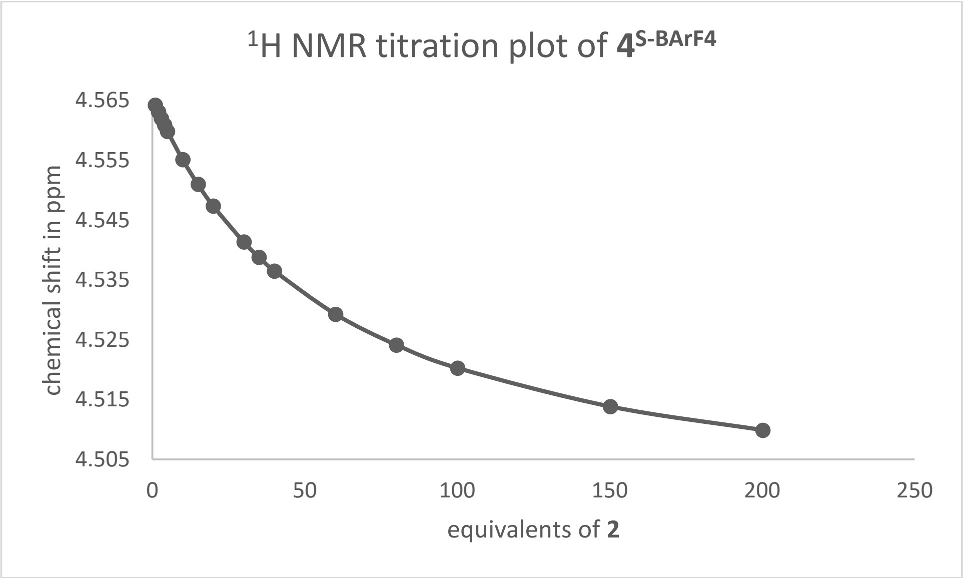

*Figure 8: ^{1}H NMR titration plot of **4**$^{S\text{-}BArF4}$ with trans-β-crotonophenone (**2**) in CD_2Cl_2.*

Table 3: ***Binding constants for the coordination of catalysts to trans-β-crotonophenone (2) in DCM-d₂.***

Entry	Host	Guest	Solvent	K [M^{-1}]
1	**4**$^{Te\text{-}BArF44}$	2	DCM-d_2	1.9
2	**4**$^{Te\text{-}BF4}$	2	DCM-d_2	4.0
3	**4**$^{Te\text{-}OTf}$	2	DCM-d_2	2.6
4	**4**$^{Se\text{-}BArF4}$	2	DCM-d_2	2.0
5	**4**$^{S\text{-}BArF4}$	2	DCM-d_2	1.8

4. Literature

[1] a) S. H. Jungbauer, S. M. Huber, *J. Am. Chem. Soc.* **2015**, *137*, 12110-12120; b) A. Dreger, E. Engelage, B. Mallick, P. D. Beer, S. M. Huber, *Chem. Commun.* **2018**, *54*, 4013-4016.

[2] P. N. Bartlett, D. C. Cook, M. W. George, J. Ke, W. Levason, G. Reid, W. Su, W. Zhang, *Phys. Chem. Chem. Phys.* **2010**, *12*, 492-501.

[3] P. Wonner, A. Dreger, L. Vogel, E. Engelage, S. M. Huber, *Angew. Chem. Int. Ed.* **2019**, *58*, 16923-16927.

[4] D. von der Heiden, S. Bozkus, M. Klussmann, M. Breugst, *J. Org. Chem.* **2017**, *82*, 4037-4043.

[5] T. V. Moskovkina, *Chem. Heterocyc. Com.* **2002**, *38*, 1190-1199.

[6] Z. Yang, C. Liu, Y. Zeng, J. Zhang, Z. Wang, Z. Fang, K. Guo, *RSC Adv.* **2016**, *6*, 89181-89184.

[7] G. Blay, I. Fernández, J. R. Pedro, C. Vila, *Synthesis* **2012**, *44*, 3590-3594.

[8] M. Bandini, M. Fagioli, P. Melchiorre, A. Melloni, A. Umani-Ronchi, *Tetrahedron Lett.* **2003**, *44*, 5843-5846.

[9] a) P. Thordarson, *Chem. Soc. Rev.* **2011**, *40*, 1305-1323; b) D. Brynn Hibbert, P. Thordarson, *Chem. Commun.* **2016**, *52*, 12792-12805.
